# Optimal delineation of single C-tactile and C-nociceptive afferents in humans by latency slowing

**DOI:** 10.1152/jn.00939.2016

**Published:** 2017-01-25

**Authors:** Roger H. Watkins, Johan Wessberg, Helena Backlund Wasling, James P. Dunham, Håkan Olausson, Richard D. Johnson, Rochelle Ackerley

**Affiliations:** ^1^Department of Physiology, University of Gothenburg, Gothenburg, Sweden;; ^2^School of Physiology and Pharmacology, University of Bristol, Bristol, United Kingdom;; ^3^University Division of Anaesthesia, Cambridge University Hospitals, NHS Foundation Trust, Cambridge, United Kingdom;; ^4^Department of Physiological Sciences, University of Florida, Gainesville, Florida;; ^5^Center for Social and Affective Neuroscience, Department of Clinical and Experimental Medicine, Linkoping University, Linkoping, Sweden; and; ^6^Laboratoire de Neurosciences Intégratives et Adaptatives (UMR 7260), Aix-Marseille Université-CNRS, Marseille, France

**Keywords:** human, microneurography, C fiber, nociceptor, low-threshold mechanoreceptor

## Abstract

C-mechanoreceptors in humans comprise a population of unmyelinated afferents exhibiting a wide range of mechanical sensitivities. C-mechanoreceptors are putatively divided into those signaling gentle touch (C-tactile afferents, CTs) and nociception (C-mechanosensitive nociceptors, CMs), giving rise to positive and negative affect, respectively. We sought to distinguish, compare, and contrast the properties of a population of human C-mechanoreceptors to see how fundamental the divisions between these putative subpopulations are. We used microneurography to record from individual afferents in humans and applied electrical and mechanical stimulation to their receptive fields. We show that C-mechanoreceptors can be distinguished unequivocally into two putative populations, comprising CTs and CMs, by electrically evoked spike latency changes (slowing). After both natural mechanical stimulation and repetitive electrical stimulation there was markedly less latency slowing in CTs compared with CMs. Electrical receptive field stimulation, which bypasses the receptor end organ, was most effective in classifying C-mechanoreceptors, as responses to mechanical receptive field stimulation overlapped somewhat, which may lead to misclassification. Furthermore, we report a subclass of low-threshold CM responding to gentle mechanical stimulation and a potential subclass of CT afferent displaying burst firing. We show that substantial differences exist in the mechanisms governing axonal conduction between CTs and CMs. We provide clear electrophysiological “signatures” (extent of latency slowing) that can be used in unequivocally identifying populations of C-mechanoreceptors in single-unit and multiunit microneurography studies and in translational animal research into affective touch. Additionally, these differential mechanisms may be pharmacologically targetable for separate modulation of positive and negative affective touch information.

**NEW & NOTEWORTHY** Human skin encodes a plethora of touch interactions, and affective tactile information is primarily signaled by slowly conducting C-mechanoreceptive afferents. We show that electrical stimulation of low-threshold C-tactile afferents produces markedly different patterns of activity compared with high-threshold C-mechanoreceptive nociceptors, although the populations overlap in their responses to mechanical stimulation. This fundamental distinction demonstrates a divergence in affective touch signaling from the first stage of sensory processing, having implications for the processing of interpersonal touch.

## Abstract (precis)

Human skin encodes a plethora of touch interactions, and affective tactile information is primarily signaled by slowly conducting C-mechanoreceptive afferents. We show that electrical stimulation of low-threshold C-tactile afferents produces markedly different patterns of activity compared with high-threshold C-mechanoreceptive nociceptors, although the populations overlap in their responses to mechanical stimulation. This fundamental distinction demonstrates a divergence in affective touch signaling from the first stage of sensory processing, having implications for the processing of interpersonal touch.

human unmyelinated c fibers responding to touch are categorized into two putative types: C-tactile (CT) afferents (Vallbo et al. 1999) and C-mechanosensitive nociceptors (CMs) (Schmidt et al. 1995, 1997), encoding positive and negative affect, respectively. It is unknown whether these are truly separate or if they form a continuum. Understanding the fundamental differences and similarities in these afferents is paramount for peripheral and central investigations into emotional touch and is especially important in pathological situations such as allodynia, where the boundaries between touch and pain signaling become less clear. CTs are identified through their mechanical sensitivity, yet there is some overlap between their responses and those from CMs (Nordin 1990; Vallbo et al. 1993, 1999), although the hedonic effects are opposite. CMs are typically identified and classified by their axonal conduction latency “profile” to repetitive electrical stimulation of the skin, based on the extent of latency slowing seen during repetitive stimulation (Schmidt et al. 1995; Serra et al. 1999).

Distinguishing C-mechanoreceptive afferents with electrical skin stimulation relies on intrinsic differences between axonal conduction, compared with understanding their sensory properties. The delivery of naturalistic mechanical stimuli during regular electrical stimulation, known as the marking technique, associates an afferent’s naturally induced and electrically induced activity. When natural stimulation increases the latency of a C fiber to electrical stimulation, conditioning activity is inferred, and the fiber is “marked” as responsive to this stimulation. The marking technique has only been validated in CMs (Schmelz et al. 1995), and afferent characterization, based on latency changes, has not yet been applied to a population of human C-mechanoreceptors, including CTs, although a case report has been made of a single low-threshold C fiber in a nerve-injured patient (Campero et al. 2011).

In animals, low-threshold C-mechanoreceptive afferents (CLTMs) have been identified in a number of species and are typically distinguished from high-threshold C-mechanoreceptive afferents (CHTMs) by their mechanical activation threshold (Bessou et al. 1971; Hoffmann et al. 2015; Kumazawa and Perl 1977; Leem et al. 1993). Studies of animal C-mechanoreceptor latency changes using electrical stimulation have produced conflicting results, with some finding clear differentiation into CHTM and CLTM afferents (Gee et al. 1996; Taguchi et al. 2010) but others finding subtle or no differences (Hoffmann et al. 2015; Hulse 2016; Obreja et al. 2010). Akin to the human literature, it is unclear whether animal C-mechanoreceptive afferents also consist of distinct populations, or how their response properties correspond.

Human C-mechanoreceptors may form a single population of mechanically responsive C-afferents, with CTs and CMs being those with greater and lesser mechanical sensitivity, respectively, or form two (or more) separate populations, divisible on the basis of their mechanically and/or electrically evoked responses. Understanding fundamental divisions between C-mechanoreceptor subpopulations will enhance our understanding of the distinctions between touch and pain signaling and may facilitate selective targeting of these systems. The present study aimed at characterizing a range of C-mechanoreceptive afferents with differing mechanical sensitivities, using latency changes during electrical stimulation. Specific hypotheses were tested, based on the following questions: *1*) Can all C-mechanoreceptive afferents be identified and distinguished by the marking technique? *2*) Can they be differentiated and categorized into separate populations by profiles of latency changes during repetitive electrical stimulation? *3*) How do these responses correspond with categorization by natural stimulation across afferents with different mechanical response properties?

## MATERIALS & METHODS

The experiment was approved by the University of Gothenburg ethics committee and performed in accordance with the Declaration of Helsinki, and written informed consent was obtained. Microneurographic axonal recordings were made from the left antebrachial cutaneous nerve (lateral or dorsal branch) in 20 sessions from 19 healthy human participants (25 ± 6 yr; 9 men, 10 women). Single-unit recordings were gained from CT and CM afferents in the forearm through an insulated, high-impedance tungsten recording electrode (FHC). Nerve signals were amplified, band-pass filtered (0.2–4 kHz), and digitized (20.8 kHz) with a Power1401 and Spike2 software (CED). C-mechanoreceptive units were identified by stroking or pinching the skin innervation territory, and single-unit waveforms were identified online with a combination of threshold crossing and template matching (Spike2).

The point of maximal tactile sensitivity in the receptive field was identified, and the monofilament with minimal bending force that reliably evoked a response from the unit was determined. To assess unit responsiveness to innocuous mechanical stimulation, a soft brush was stroked slowly across the receptive field. Units were initially classified as CT if they responded to the brush stimulus, typically with a burst of spikes, and CM if they did not respond (Vallbo et al. 1999). Spike shape measurements were obtained for all units identified with marking stimulation and were generated by taking the first spike evoked by sensory stimulation after a period of >10 s without stimulation during the first 5 min of recording.

### 

#### Receptive field electrical stimulation of physiologically characterized units.

The effect of electrical receptive field stimulation on a single unit’s axonal conduction velocity was explored with three protocols. Constant-current electrical stimulation (0.5-ms pulse width) was delivered through two uninsulated tungsten electrodes (FHC) inserted obliquely into the skin and advanced to within ~1 mm of the point of maximum tactile sensitivity on the skin surface, with a reference electrode inserted in the same manner 5–10 mm medial/lateral to this (Schmidt et al. 1995). The electrical threshold of the unit (T) was determined, and stimulus intensity was set at 2T. Three different electrical stimulation protocols were employed: *1*) marking stimulation, to investigate the dependence of latency shifts on spikes evoked by natural stimulation; *2*) a standard 2-Hz protocol, which is effective at identifying CMs; and *3*) high-frequency stimulation, to examine stimulus entrainment and latency shifts at physiological discharge frequencies and near the refractory period.

Marking stimulation consisted of repetitive 0.25-Hz electrical stimulation (Schmelz et al. 1995) delivered to the receptive field, with concurrent mechanical stimuli with suprathreshold monofilaments or brushing between electrical stimuli. If mechanical activation caused an increase in response latency to electrical stimulation, the electrically evoked responses were presumed to originate from the physiologically characterized unit and further electrical stimulation was performed. After marking, units were not electrically or mechanically stimulated for >2 min, to allow for the recovery of spike conduction to baseline levels. Conduction velocity was estimated by dividing the distance from the receptive field to the recording electrode insertion site by the latency of the first spike evoked after this period.

Two-hertz electrical stimulation, over a period of 3 min, was used (Obreja et al. 2010; Serra et al. 1999), followed by 10 pulses of 0.25-Hz stimulation, with >2 min for recovery before further electrical stimulation.

High-frequency electrical stimulation consisted of four pulses at frequencies of 10, 20, 50, 100, or 200 Hz, with 30-s recovery and three repeats of all frequencies (with the exception of 10 and 20 Hz, which were given once). All trains were delivered in a randomized order.

#### Microneurography data analysis.

Analysis of microneurography data was performed in Spike2 with off-line template matching. The presence of spikes and their latency (to spike negative-going peak) were analyzed with a custom-written script. Latency changes were represented as a percentage relative to the first spike elicited by each train of stimulation. Analyses of marking data involved counting the number of spikes evoked by a mechanical stimulus in the period between two electrical stimuli, with the latency change expressed as a percentage of the response before tactile stimulation. Marking responses that caused accumulative latency shifts or collisions were not included in the analyses.

One unit (CM) was excluded from the 2-Hz analysis because of stimulation failures, and one CM unit showed latency jumps (abrupt shifts from a distinct response latency) during high-frequency stimulation, presumably reflecting activations of another axonal branch and causing longer-latency responses. Occasional latency jumps were observed in other units (see Fig. 2*C*) during the marking and 2-Hz stimulation, which were excluded from the latency analysis. All statistical analyses were conducted in GraphPad Prism, where *P* < 0.05 was considered significant, by two-tailed parametric tests. Linear regressions were compared by ANCOVA.

## RESULTS

### 

#### Classification of C-mechanoreceptive units from their physiological properties.

**Fig. 1.**
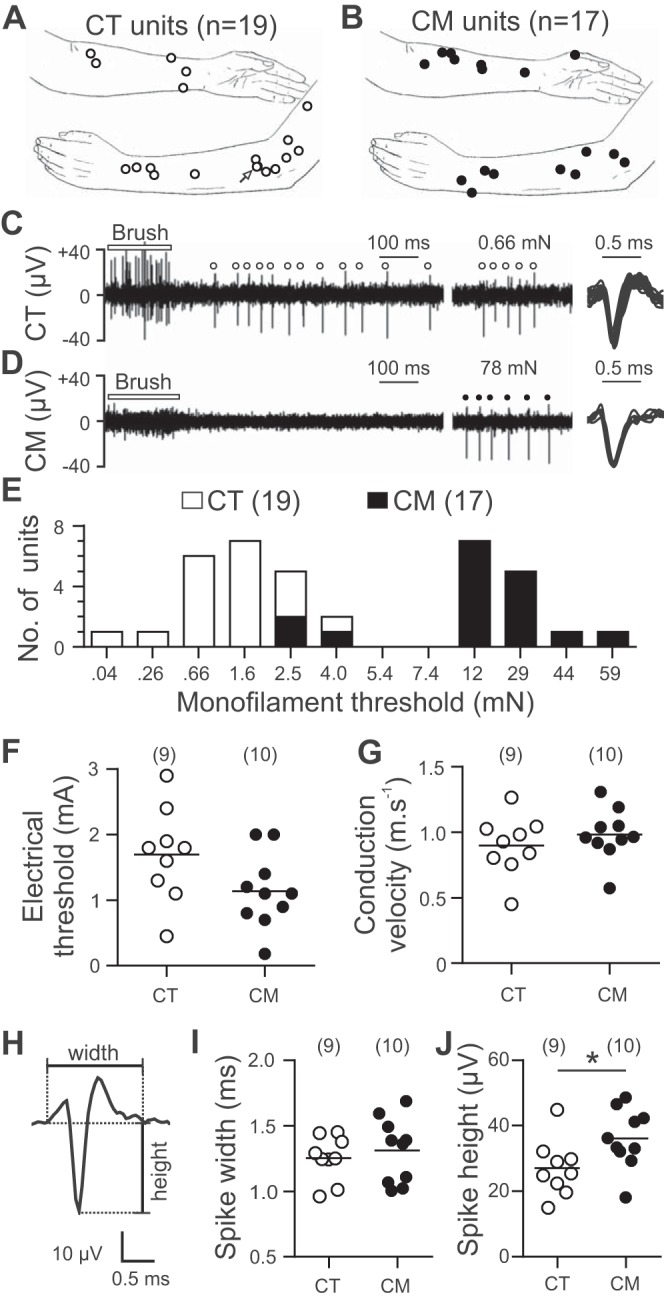
Physiological properties of CM and CT afferents. *A* and *B*: indicators of receptive field locations of CT (*A*) and CM (*B*) units on the arm. Arrow indicates 2 CT units with overlapping receptive field locations. *C* and *D*: responses to brushing and suprathreshold indentation from a CT (monofilament threshold 0.04 mN) (*C*) and a CM (monofilament threshold 12 mN) (*D*) and overlaid spikes from all displayed responses. Spike times are marked above the trace. Multiunit discharges occurred in myelinated afferents during the brush stimulation (*C* and *D*), with a long-latency response in the CT unit, with a typical afterdischarge outlasting the stimulus (*C*), and no response in the CM unit (*D*). *E*: a bimodal distribution of monofilament thresholds was seen, but with some overlap between CTs and CMs. *F* and *G*: intracutaneous electrical thresholds (*F*) and conduction velocities (*G*) were not significantly different between CTs and CMs. *H*: spike shape measurements. *I* and *J*: there was no significant difference between CT and CM spike widths (*I*), but spike amplitude was significantly smaller in CTs (*J*; *P < 0.05).

Single-unit recordings were obtained from 36 C-mechanoreceptive afferents. On the basis of their responses to brush stroking (Fig. 1, *C* and *D*), 19 were classified initially as CT and 17 as CM, with their receptive fields indicated in Fig. 1, *A* and *B*. All units were identified as slowly conducting based on their clear, delayed responses to mechanical stimulation (Fig. 1*C*). Mechanical activation thresholds fell into two broad populations (Fig. 1*E*), and the population of C-mechanoreceptors was predominantly separated into brush-responsive low-threshold (<5 mN) CTs (Fig. 1*C*) and brush-unresponsive high-threshold (>10 mN) CMs (Fig. 1*D*). Notably, three CM afferents initially appeared to be low-threshold units on the basis of mechanical threshold obtained (<5 mN), although they lacked vigorous responses to brushing. These low-threshold CM afferents had a single point of high mechanical sensitivity, and one afferent was able to generate a single spike to the brush stimulus but not the typical burst of >10 spikes seen in CTs (Fig. 1*C*). Nine CT and 10 CM single-unit recordings were maintained to allow identification by electrical stimulation of the skin and the marking technique as well as their spike shape. The threshold for electrical stimulation, conduction velocity, and spike width were not significantly different between CTs and CMs (Fig. 1, *F*, *G*, and *I*, respectively), although CTs had significantly smaller spike amplitudes (*P* < 0.05; Fig. 1*J*).

#### C-mechanoreceptor classification using conditioning of electrical responses with mechanical stimulation (marking).

**Fig. 2.**
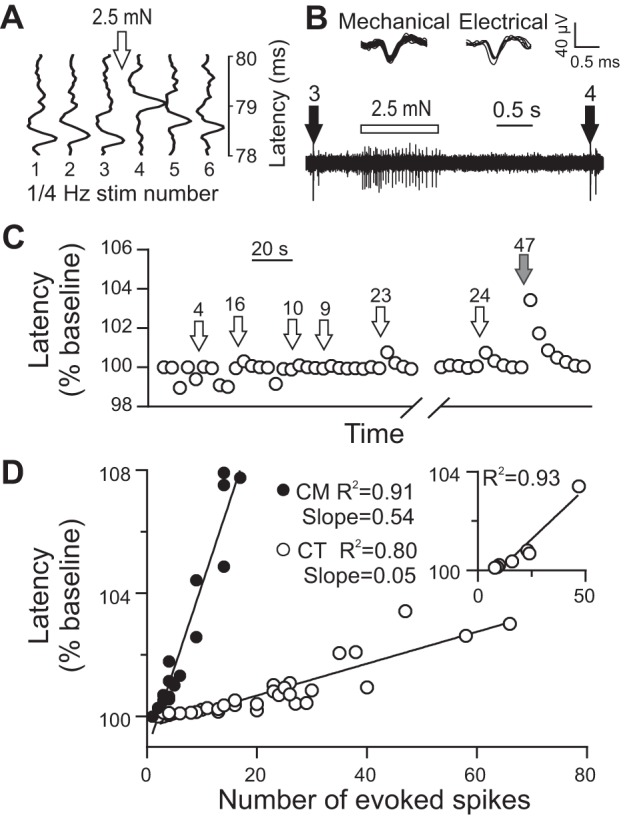
Marking responses in CT and CM afferents. *A*: during electrical stimulation at 0.25 Hz, physiologically stimulating the CT unit (between electrical *stimuli 3* and *4*) increased the latency to electrical stimulation. The relative timings of sensory and electrical stimuli are shown on the original trace in *B*. *C*: extent of latency changes to 7.4-mN monofilament (open arrows) and brush (filled arrow) stimulation in a CT unit is related to the number of evoked spikes (numbers above arrows). Some shorter-latency spikes were seen, presumably originating from a shorter axonal branch, and were excluded from the analysis of latency shifts. *D*: number of evoked spikes correlated significantly with the observed latency changes in both CT (*P* < 0.001) and CM (*P* < 0.001) units, with significantly different slopes (*P* < 0.001). *Inset*: the same data for the unit shown in *C* (*P* < 0.001). Data presented are from 36 markings from 8 CTs and 16 markings from 8 CMs.

The marking technique (Fig. 2, *A* and *B*) provided positive identification of all C-mechanoreceptive units. Spike latency changes to electrical stimulation were seen when delivering natural stimulation concomitantly. The number of spikes evoked by natural stimulation was strongly correlated with the observed change in latency to electrical stimulation for both CTs (slope = 0.05, *R*^2^ = 0.80, *P* < 0.001; 36 markings from 8 units; see Fig. 2, *C* and *D* for an individual CT unit and the group, respectively) and CMs (slope = 0.54, *R*^2^ = 0.91, *P* < 0.001; 16 markings from 8 units; Fig. 2*D*). The dependence of latency changes on the number of evoked spikes was strikingly diverse between CT and CM units, with a significant difference in the slopes of the linear regressions (*P* < 0.001). The slopes show that each additional spike caused a latency shift of 0.5% in CMs but only a 0.05% shift in CTs.

#### Classification of C-mechanoreceptive units by latency changes during 2-Hz electrical stimulation.

**Fig. 3.**
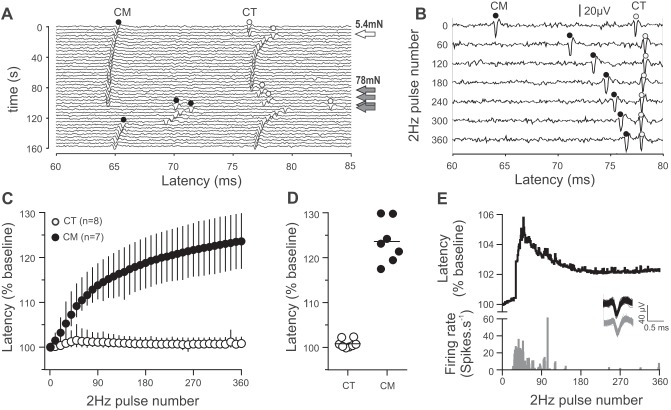
Latency changes during 2-Hz stimulation in CM and CT afferents. *A*: marking responses [indicated with filled (CM) and open (CT) circles] show that both the CT and the CM responded to the 78-mN monofilament and the CT neuron responded to the 5.4-mN monofilament. *B*: raw records of units shown in *A* to 2-Hz electrical stimulation, with every 60th electrical stimulation displayed. *C*: latency changes in all CT and CM units during 2-Hz electrical stimulation. For clarity, every 10th stimulation is displayed; symbols and error bars denote mean and range, respectively. *D*: scatterplot of latency changes at the 360th pulse during 2-Hz stimulation in CTs and CMs; lines indicate means. *E*: nonelectrically evoked spikes during 2-Hz electrical stimulation induce a latency increase that recovers as the firing stops. *Inset*: traces show overlaid electrically evoked (black) and nonelectrically evoked (gray) spikes.

During 2-Hz electrical stimulation, a dramatically different pattern of spike latency changes was seen between two populations of C-mechanoreceptors, corresponding to CT and CM units (Fig. 3*C*). An example of a simultaneous CT and CM recording is shown in Fig. 3, *A* and *B*; in this recording the CT readily responded to low-intensity (5.4 mN) monofilament stimulation, and both units responded to a stronger (78 mN) monofilament stimulation (Fig. 3*A*). The CT showed a 0.5% latency increase during 2-Hz stimulation, whereas the CM showed a 20% increase (Fig. 3*B*). This difference was reflected in the population, where the slowing after 40 pulses delivered at 2 Hz could be used to unequivocally classify all afferents in the sample and the difference after 360 pulses was even more pronounced, with little variance seen in the CT population (Fig. 3, *C* and *D*). Both CMs with low mechanical thresholds (2.5 mN) showed typical CM latency changes (>20%) during 2-Hz stimulation. A subpopulation of CTs (*n* = 4) generated additional spikes that were not time-locked to the 2-Hz electrical stimulus, and the most pronounced example of this is shown in Fig. 3*E*. In these units, there was a delay of 15–60 pulses before the additional spike burst generation. All units recovered and showed a plateau in latency after the additional firing stopped, and the only two CTs showing >1% latency change during the 2-Hz stimulation were the CTs with most pronounced firing (>10 spikes/s).

#### Classification of C-mechanoreceptive units through their response to high-frequency electrical stimulation.

**Fig. 4.**
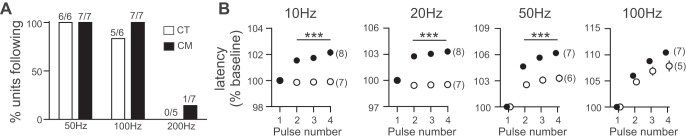
Latency changes during high-frequency stimulation in CM and CT afferents. *A*: similar proportions of CT and CM units followed all the pulses of the higher-frequency electrical stimulation. *B*: CT units showed significantly less latency increase than CM units during stimulation at 10–50 Hz (***P < 0.001 in all cases). Symbols and error bars indicate means ± SE, with the number of units in parentheses; error bars are masked by the symbols in most cases.

Few differences were observed in the ability of CT and CM units to follow high-frequency (>50 Hz) electrical stimulation (Fig. 4*A*), and the only afferent following all pulses at 200 Hz was a CM with a low mechanical threshold. There were significantly smaller latency increases in CTs compared with CMs during 10-, 20-, and 50-Hz stimulation (all *P* < 0.001), but no significant differences were observed at 100 Hz. CTs showed significantly less accumulative slowing (the difference between the second and third pulse and between the third and fourth pulse) at 10, 20, and 50 Hz (all *P* < 0.01) but only between the third and fourth pulse at 100 Hz (*P* < 0.05).

## DISCUSSION

We found that human C-mechanoreceptive afferents could be separated unequivocally into two populations on the basis of their responses to electrical stimulation. Mechanical stimulation produced some inconsistencies with the classification. Brush stimulation separated the C-mechanoreceptors into a population of CTs (brush responsive) and CMs (brush unresponsive), although a few CMs responded weakly to the brush. Mechanical monofilament thresholds separated the populations, where CTs had thresholds of <5 mN but a few CMs fell into this range. These findings demonstrate the similarities and differences between properties of C-mechanoreceptive afferents; therefore care must be taken when classifying C-mechanoreceptors.

The marking technique was sensitive enough to detect the latency shift evoked by a single spike in CMs (Schmelz et al. 1995), but to achieve a detectable latency shift in a CT 10 spikes need to be evoked. This suggests that CT thresholds and weak responses cannot be assessed with this technique, although suprathreshold brush responses may be characterizable (Fig. 2*C*).

CT responses to 2-Hz electrical stimulation were distinct from CMs and all other C-fiber afferent and efferents (Obreja et al. 2010; Serra et al. 1999). There are large differences in mechanical activation thresholds between human CTs (Nordin 1990; Vallbo et al. 1993, 1999) and animal CLTMs (Leem et al. 1993; Seal et al. 2009); thus electrical stimulation seems superior for classification and comparison. The previously unidentified “type 3” fibers with latency slowing <1% during 2-Hz stimulation in human and rat recordings (George et al. 2007; Serra et al. 1999) likely correspond to CTs and an animal equivalent of CTs, respectively. Repetitive 2-Hz stimulation may be used to compare CTs with genetically identified CLTMs (Seal et al. 2009; Vrontou et al. 2013) and other novel CLTM populations (Djouhri 2016). Half of the CTs recorded produced additional discharges during 2-Hz stimulation; the cause of this is unclear. Afferents showing this pattern may be detectable as CTs in multiunit recordings and may form a subpopulation of CT afferents. Animal CLTMs slowing >10% with 2-Hz stimulation (Hoffmann et al. 2015; Hulse 2016; Obreja et al. 2010) likely correspond to a population of CM fibers with low mechanical thresholds (cf. the low-threshold CMs found here).

Latency changes in mechanosensitive C fibers reflect changes in mechanical thresholds and excitability (De Col et al. 2012). We predict that the smaller latency changes in CTs than in CMs during stimulation at physiological rates (10–50 Hz) allow the generation and maintenance of higher spiking rates to mechanical stimulation, enabling the rates observed in CTs in response to mechanical stimuli (up to 100 spikes/s; Vallbo et al. 1999). The physiological upper firing capability appears similar between CMs and CTs from the minimal differences found between entrainment and latency changes at higher frequencies. Absolute refractory period may be governed by similar processes in these two populations, despite repetitive conditioning revealing different axonal mechanisms.

Disparities in conduction latency changes between CTs and CMs during repetitive stimulation are likely produced by differences in the expression of ion channels or ionic transporting mechanisms. A difference in voltage-gated ion channels is suggested by the narrower somatic spike widths in CLTMs than in CHTMs in the rat (Fang et al. 2005), and we find that CTs had significantly smaller spike amplitudes (Fig. 1*H*). The expression of voltage-gated sodium channel subtypes, which play a role in C-fiber latency changes (De Col et al. 2008; Kankel et al. 2012; Obreja et al. 2012), may be different between CTs and CMs, for example, with CTs expressing a subtype less prone to slow inactivation.

Identifying and characterizing fundamental divisions between C-fiber populations in the periphery provides opportunities to selectively modulate affect at the first stage of encoding. Targeting receptors at the periphery is particularly advantageous to avoid systemic side effects. Thus abhorrent pain signaling, as seen in various neuropathies (Kleggetveit et al. 2012; Serra et al. 2012, 2014), may be treated directly in the periphery without altering affective touch. Furthermore, it may be possible to explore the role of CTs in pain and enhance pleasure in touch, without impacting on normal nociceptive functioning. This has implications for the function of CTs in pathologies, where they are proposed to contribute to mechanical (Liljencrantz et al., 2013; Nagi et al. 2011) and cold (Samour et al. 2015) allodynia, as well as understanding their role in gating pain (Krahé et al. 2016; Liljencrantz et al. 2014). The propensity of some low-threshold CMs to fire in response to gentle mechanical stimulation must be considered in future human behavioral and brain imaging studies, especially in pathological situations where response properties may be altered. Further investigations into the potential subgroups of CTs and CMs are warranted to understand their roles in signaling affective touch and pain, which has implications for the targeting of specific types of C fibers in translational animal studies.

The marking technique and 2-Hz electrical stimulation protocols provide unequivocal separation of C-mechanoreceptors into putative gentle touch-signaling CT and nociceptive-signaling CM populations; however, we show overlap in some C-mechanoreceptive afferent physiological response properties. Our findings enable the unambiguous identification of CTs, which can be applied to future human multiunit microneurography recordings and provides a framework for comparing animal CLTMs to human CTs. The intrinsic axonal conduction differences highlight the fundamental differences between peripheral afferents signaling positive (CT) and negative (CM) affective touch. The underlying mechanisms may be pharmacologically targetable for control over the selective modulation and excitability of CT and CM firing, especially in pathological situations involving tactile dysfunction, such as allodynia.

## GRANTS

This work was supported by the Swedish Medical Research Council (Grants 62X-3548 to J. Wessberg and 2010-2607 to H. Olausson), Sahlgrenska University Hospital (ALFGBG Grant 3161 to J. Wessberg), and the Medical Research Council, UK (Centenary Award Fellowship Grant MR/J500446/ to R. H. Watkins).

## DISCLOSURES

No conflicts of interest, financial or otherwise, are declared by the author(s).

## AUTHOR CONTRIBUTIONS

R.H.W., J.W., H.B.W., J.P.D., H.O., R.D.J., and R.A. conceived and designed research; R.H.W., J.W., H.B.W., J.P.D., and R.A. performed experiments; R.H.W., J.P.D., and R.A. analyzed data; R.H.W., J.W., H.B.W., J.P.D., H.O., R.D.J., and R.A. interpreted results of experiments; R.H.W. and R.A. prepared figures; R.H.W., J.W., H.B.W., J.P.D., H.O., R.D.J., and R.A. drafted manuscript; R.H.W., J.W., H.B.W., J.P.D., H.O., R.D.J., and R.A. edited and revised manuscript; R.H.W., J.W., H.B.W., J.P.D., H.O., R.D.J., and R.A. approved final version of manuscript.
